# The Role of Androgen Hormones in Early Follicular Development

**DOI:** 10.1155/2014/818010

**Published:** 2014-04-10

**Authors:** Catiele Garcia Gervásio, Marcelo Picinin Bernuci, Marcos Felipe Silva-de-Sá, Ana Carolina Japur de Sá Rosa-e-Silva

**Affiliations:** Sector of Human Reproduction, Department of Gynecology & Obstetrics, Ribeirão Preto Medical School, University of São Paulo, Avenida Bandeirantes, 3900 Ribeirão Preto, SP, Brazil

## Abstract

*Background*. Although chronic hyperandrogenism, a typical feature of polycystic ovary syndrome, is often associated with disturbed reproductive performance, androgens have been shown to promote ovarian follicle growth in shorter exposures. Here, we review the main effects of androgens on the regulation of early folliculogenesis and the potential of their application in improving follicular *in vitro* growth. *Review*. Androgens may affect folliculogenesis directly via androgen receptors (ARs) or indirectly through aromatization to estrogen. ARs are highly expressed in the granulosa and theca cells of early stage follicles and slightly expressed in mature follicles. Short-term androgen exposure augments FSH receptor expression in the granulosa cells of developing follicles and enhances the FSH-induced cAMP formation necessary for the transcription of genes involved in the control of follicular cell proliferation and differentiation. AR activation also increases insulin-like growth factor (IGF-1) and its receptor gene expression in the granulosa and theca cells of growing follicles and in the oocytes of primordial follicles, thus facilitating IGF-1 actions in both follicular recruitment and subsequent development. *Conclusion*. During the early and intermediate stages of follicular maturation, locally produced androgens facilitate the transition of follicles from the dormant to the growing pool as well as their further development.

## 1. Introduction

The development of ovarian follicles begins during fetal life with the transformation of primordial germ cells into oocytes enclosed in structures called follicles [1, 2]. Some of these follicles are recruited to start a long progress of growth and differentiation, during which the proteins required for oocyte maturation are progressively synthesized and accumulated [3, 4]. All events related to follicular development are regulated by appropriate signals originating from the growing oocyte itself and from the somatic cells that surround it [5, 6] and also by complex interactions between gonadotropin hormones, sex steroids, and diverse growth factors [7, 8].

The sex steroids produced by follicular cells are known to play major roles in the regulation of ovarian function. When present in the systemic circulation, these steroids actively participate in the regulation of pituitary gonadotropin secretion, and when present in the ovarian microenvironment, they act as important paracrine factors for the maintenance of follicular development [9]. Although much of the information about the role of sex steroids in ovarian functioning has been obtained in studies directed at the action of estrogens [10–12] and progestogens [13–15], increasing attention is being devoted to the action of androgen hormones because the activation of the androgen receptor located in follicular cells [16, 17] modulates the expression and activity of important genes for the maintenance of ovarian follicle development [17–19].

Additional evidence of the action of androgens in the regulation of folliculogenesis has arisen from* in vitro *studies showing that various androgens, including testosterone, androstenedione, and dihydrotestosterone, can stimulate the growth and development of ovarian follicles in mammals [20–22]. The reduction of reproductive function and the development of premature ovarian failure in mice with the nonselective deletion of the androgen receptor gene [23–25] support the hypothesis of the involvement of androgens in the regulation of follicular development. Reinforcing these findings, mice carrying this deletion show the impaired* in vitro *development of preantral follicles [26]. Additionally, a more pronounced expression of the androgen receptors has been reported to occur in preantral follicles [27, 28], suggesting the major action of androgens during the initial stages of folliculogenesis.

From a clinical point of view, polycystic ovary syndrome (PCOS) is a nosologic entity that affects approximately 5–10% of women of reproductive age and is characterized by increased ovarian androgen production and chronic anovulation. The excessively androgenic microenvironment of the ovary is believed to have a negative impact on follicular development, which, in addition to LH hypersecretion, promotes follicle stagnation in the early stages of development (initial antral), inhibiting the development of a dominant and ovulatory follicle and leading to chronic anovulation and infertility. Among the various therapeutic alternatives for infertile patients with this diagnosis is the* in vitro* maturation of follicles obtained from their ovaries without previous induction. The results of this technique are limited in terms of the reproductive outcome, likely due to the impaired quality of the oocytes developed in hyperandrogenic environments [29]. Another therapeutic possibility in these cases is* in vitro* fertilization procedure, which shows that patients with PCOS, despite a larger number of oocytes, have similar pregnancy rates. This suggests a poorer utilization of the oocytes obtained, again indicating impaired oocyte quality [30].

Therefore, there is evidence in the literature of the participation of androgens in follicular development both as essential adjuvants and as harmful agents when present in excessive amounts. This indicates the relevance of understanding the role of androgens in the regulation of folliculogenesis, as well as the possibility of recreating* in vitro* conditions capable of guaranteeing the full growth of ovarian follicles to mimic as much as possible the* in vivo* intrafollicular environment [31]. On this basis, the objective of the current review was to present more relevant data regarding the involvement of androgens in the regulation of folliculogenesis and to provide information for the design of future culture strategies that can be used to promote the* in vitro* development of ovarian follicles.

## 2. Androgen Synthesis in the Ovary

**Figure 1:**
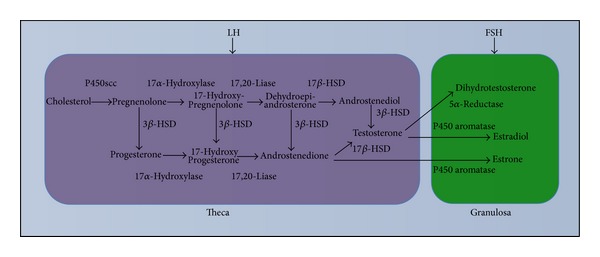
Schematic view of the production of androgen hormones by ovarian follicular cells according to the two-cells-two-hormones model. In the theca cells, the androgens (androstenedione and testosterone) are produced in response to the luteinizing hormone (LH) stimulus. After diffusing towards the granulosa cells, the androgens are converted to estrogens (estradiol and estrone) by the enzyme aromatase under the action of the follicle stimulating hormone (FSH). P450scc, enzyme responsible for the cleavage of the lateral chain of cholesterol; 3*β*-HSD, 3*β*-hydroxysteroid dehydrogenase; 17*β*-HSD, 17*β*-hydroxysteroid dehydrogenase.

The androgens androstenedione, testosterone, and dihydrotestosterone are primarily synthesized from cholesterol and are produced by the ovary in a sequential manner together with other sex steroids, progestogens, and estrogens, with each steroid serving as a substrate for the subsequent one in a cascade of events known as steroidogenesis [32–34]. The classical two-cells-two-hormones model describes the role of follicular cells (theca and granulose) and of gonadotropins (follicle stimulating hormone (FSH) and luteinizing hormone (LH)) in steroid synthesis and secretion in the ovary, with emphasis on the cooperation of the two cell types that are necessary for androgen production. In general, the androstenedione synthesized from progestogens is converted to testosterone by the action of the enzyme 17*β*-hydroxysteroid dehydrogenase in theca cells under the LH stimulus, and the produced androgen is passively transported to the granulose cells where it is converted in estrogen by the action of aromatase under the FSH stimulus [35] (Figure 1).

Due of this conversion of testosterone in estradiol in the granulosa, many of the actions of androgens on the growth and differentiation of ovarian follicles can be indirectly mediated by the action of androgens as precursors in the biosynthesis of estrogens. Although the actions of estrogens on the ovary are well known in terms of the pattern of expression and function of estrogen receptors [36], little is known about the direct involvement of androgens in terms of their interaction with the specific receptor in the regulation and maintenance of folliculogenesis.

## 3. Expression of the Androgen Receptor in the Ovary

The cellular actions of androgens require the binding and activation of the specific ligand receptor called the androgen receptor (AR) [37]. Both the protein and messenger RNA of AR have been detected in the ovary of various mammalian species, such as rodents [38, 39], cattle [40, 41], sheep [42], swine [43, 44], non-human primates [16, 17], and humans [45, 46]. Although most of these studies have indicated that granulosa cells are the predominant sites of AR expression, theca and ovarian stromal cells also express AR [20, 47, 48]. In oocytes, AR expression exhibits an evolutive profile that is highly expressed in amphibians [49], moderately expressed in rodents [20], little expressed in ruminants [42, 44], and incipient or absent in non-human primates and in humans [17, 50–52].

In rodents and primates, AR expression appears to be regulated along follicular development, being increased in ovaries containing a larger number of preantral and antral follicles of small diameter and reduced in ovaries containing periovulatory follicles [53, 54]. The analysis of the expression in isolated follicles shows that, in these species, follicles in the early stages of development express a larger number of AR than those in more advanced stages (Table 1, ∗) [20, 55]. Additionally, a differential gradient of AR expression is noted in mature follicles, which are little expressed in mural granulosa cells and are highly expressed in cumulus cells [56]. The profile of mRNA and the protein of AR in different follicular classes are presented in Table 1.

## 4. Mechanisms of Action of the Androgens

Like all steroid hormones, the androgens primarily exert their functions by binding and activating specific nuclear receptors that trigger the intracellular events responsible for the beginning of the transcription of target genes [57, 58]. Additionally, the androgens can also exert their effects by interacting with receptors located on the cell membrane to perform rapid, non-genomic actions involved in the activation of various transcription factors [59–61]. Thus, the activated ARs transcriptionally regulate the expression of a selected group of genes via direct or indirect association with the regulatory regions of (enhancer/promoter) upstream elements [37, 62, 63]. Although several autocrine and/or paracrine factors involved in the regulation of the development of ovarian follicles have been described [6, 64, 65], only some genes responsible for the transcription of these factors have been tested as AR targets. Particularly important among them are the genes related to FSH receptors, insulin-like growth factor-1 (IGF-1), and aromatase enzyme [18, 19, 66, 67]. The main findings regarding the direct action of androgen hormones on the* in vivo* and* in vitro* control of follicular development in mammals are based on the transcriptional actions of AR in follicular cells. As reported for AR expression in the oocytes, the physiological role of androgens in oocyte maturation appears to have been lost over evolution, being phylogenetically replaced by the action of gonadotropins [68].

## 5. Role of Androgens in Early Follicular Development

### 5.1. *In Vivo* Effects

Although the effects of chronic exposure to high androgen concentrations during the prenatal or postnatal period in different mammalian species are extensively known and are correlated with irregularities of the reproductive cycle and changes in the ovarian morphology in patients with a diagnosis of PCOS [69], few studies have been designed to assess the effects of short-term exposure to low androgen concentrations. The main results obtained in studies evaluating the effects of the* in vivo* administration of androgen hormones on early follicular development in different species are listed in Table 2 and described below.

**Figure 2:**
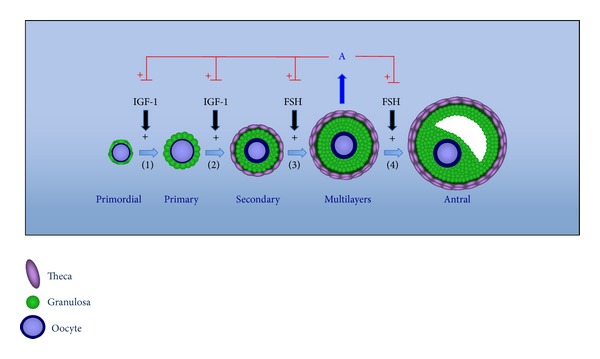
Schematic presentation of the action of androgen hormones on early follicular development. The androgens produced by the growing follicles can promote the transition from (1) primordial to primary follicles and (2) from primary to secondary follicles through the amplification of the actions of the IGF-1 system and can also amplify the actions of follicle stimulating hormone (FSH) on the promotion of the subsequent growth of (3) preantral and (4) antral follicles.

Subcutaneous implants for the controlled release of low androgen doses have been used as an efficient tool for the study of the effects of short-term androgen exposure using experimental animal models. In non-human primates, subcutaneous implants containing different doses of testosterone promoted an expressive increase in follicular recruitment, growth, and survival [70]. These effects appear to be mediated by a local amplification of the action of both IGF-1 and FSH because exposure to testosterone induced an increase in IGF-1, IGF-1 receptor, and FSH receptor mRNA in the ovaries of these animals [18, 19, 66]. The increase of follicular recruitment was positively correlated with increases in IGF-1 and IGF-1 receptor mRNA in the oocytes of primordial follicles, suggesting an indirect action of androgens via the IGF-1 system on follicular activation [19]. All of the effects on both ovarian morphology and the IGF-1 and FSH system induced via exposure to testosterone were fully replicated when subcutaneous implants containing the nonaromatizable androgen dihydrotestosterone were used, showing that the effects of androgens are mediated by AR and not by conversion to estrogens. Similarly, the* in vitro* treatment of granulosa cells obtained from the antral follicles of swine with dihydrotestosterone increases the action of IGF-1 as a stimulus to cell proliferation, as well as the effect of growth and differentiation factor 9 (GDF9) in the presence of IGF-1 [71, 72], both of which are effects that are blocked by the presence of the AR antagonist hydroxyflutamide. The positive correlation between the expression of the AR gene and the proliferation of granulosa cells and follicular growth further supports the hypothesis of the involvement of androgens in follicular growth via AR [17] (Figure 2).

In other mammalian species, short-term exposure to low androgen doses was also related to increased follicular recruitment and growth. In swine, the intramuscular administration of dihydrotestosterone during the first 3 days of the early follicular phase and during the last 3 days of the late follicular phase of the reproductive cycle significantly increased the ovulatory rate of these animals [73]. The administration of 10x diluted doses administered from the 13th day of the estrous cycle to the next estrus promoted a significant increase of mRNA expression of the FSH receptor in periovulatory follicles, suggesting that the increased ovulatory response detected in androgenized animals is in fact related to the increased sensitivity of ovarian follicles to the gonadotropic action induced by the androgen. In rodents, the administration of subcutaneous implants containing dihydrotestosterone promoted the increased expression of FSH receptor mRNA in preantral follicles [74]. Additionally, the potential for the* in vitro* development of preantral follicles isolated from the ovaries of androgenized animals was superior to that of nonandrogenized follicles, showing that in this species the androgens also promote follicular growth via increased gonadotropic sensitization.

Taken together, these findings suggest that the* in vivo* action of androgens via AR may regulate both the expression and the action of one or more of the ovarian growth factors necessary for the regulation of follicular recruitment and growth. It should be noted that no* in vivo* studies have been conducted on the human species because of the limitation represented by the side effects that this practice could cause in women exposed to systemic androgens. A satisfactory model for these evaluations in humans is represented by the study of oocytes obtained from patients with PCOS.

In 1991, Cha et al. [75] reported the first pregnancy resulting from* in vitro* maturation (IVM); in 1994, Trounson [76] first reported a successful pregnancy process using oocytes aspirated from nonstimulated patients with PCOS. Since then, various studies have performed IVM without stimulation in patients with PCOS with the objective of obtaining pregnancy rates similar to those of fertile women [75, 77–79] and to assess the efficacy of IVM and fertilization in nonstimulated patients.

In the studies cited above, immature oocytes were aspirated from infertile patients between days 6 and 14 of the cycle with the aid of transvaginal ultrasound, and those of normal morphology were placed in culture for 24–48 hours; only those with extrusion of the first polar body were submitted to intracytoplasmic sperm injection (ICSI), and transfer was performed 2 to 3 days after ICSI [75, 77–79]. In the cited reports, fertilization rates ranged from 62% to 75.3%, and cleavage rates ranged from 81.4% to 95%, which are in agreement with data reported by Trounson et al. [76]. The pregnancy rates reported by Zhao et al. [78] (40%), Holzer et al. [77] (50%), and Zhao et al. [79] (40%) are similar to those of patients with unknown causes of infertility, although Cha et al. [75] and Bos-Mikich et al. [80] reported lower pregnancy rates in PCOS patients (27.1% and 32%, resp.). According to Zhao et al. [79], ovarian stimulation is unnecessary; they obtained pregnancy rates similar to those of patients with stimulated cycles (40%). A similar result was obtained by Ge et al. [81], who showed that the addition of hCG does not contribute to oocyte maturation. The advantages of nonstimulation are many and include the prevention of ovary hyperstimulation syndrome induced by gonadotropin use, reduction of costs, shorter and continuous treatment cycles, and the prevention of a series of other long-term complications such as hormone-dependent neoplasias [75, 79] (Table 3).

According to Das et al. [82], the anti-Mullerian hormone (AMH) plays a role in PCOS because its values are increased in affected patients compared with control ovulatory women (466 ng/mL and 78 ng/mL, resp.,* p* = 11 and 8, resp.). It was also observed that early antral and preantral follicles express AMH, which is absent in primordial and in atresic follicles [82]. These authors speculated about the influence of high androgen levels on the elevation of AMH values in patients with PCOS.

The relationship between GDF9 and bone morphogenetic protein (BMP15) factor has been studied in the oocytes and granulosa cells of patients with PCOS because these factors play a crucial role in follicle development, ovulation, oocyte maturation, and embryo development. It has been reported that GDF9 and BMP15 are not expressed in patients with PCOS, with a consequent later impairment of cytoplasm maturation and poor oocyte quality, whereas they are expressed in normal ovulatory women [83, 84].

### 5.2. *In Vitro* Effects

The development of* in vitro* culture systems able to guarantee the growth and differentiation of isolated ovarian follicles represents a valuable tool for the study of the direct effects of androgens on folliculogenesis. Various* in vitro* systems have been employed for the culture of preantral follicles in various mammalian species, such as cattle [85], goats [86], non-human primates [66], and humans [87]. However, the production of healthy offspring from oocytes of preantral follicles matured* in vitro* has been reported only in mice thus far [88]. The main results obtained in studies aiming to evaluate the* in vitro* effects of androgens on early follicular development in different species are listed in Table 4 and described below.

The* in vitro* treatment of mouse ovarian follicles with AR antagonists (hydroxyflutamide and bicalutamide) reduced follicular growth during the preantral phase, as well as the meiotic maturation of the enclosed oocyte [20], suggesting the importance of androgen action in follicle maturation. The inability of preantral follicles to develop* in vitro* to preovulatory stages in the presence of antiandrogen antibodies [21] supports the hypothesis that the actions mediated by ARs are important for the early stages of follicular growth. In the same study, the addition of the AR antagonist casodex inhibited the positive effect of FSH on follicular growth, which was completely reversed when dihydroxytestosterone was added, revealing a joint action of androgens and FSH on follicular development. Additionally, the increased survival and growth of preantral follicles in the presence of androstenedione [89] represents further evidence of the positive action of androgens on follicular development. In the same study [21], the addition of antiestrogen antibodies and of estradiol receptor antagonists (ICI 182, 780) did not modify the positive effects of androstenedione on follicular growth, confirming the direct action of androgens on the development of preantral follicle maintenance.

The supplementation of the culture medium with other androgen hormones, that is, dihydrotestosterone, testosterone, DHEA, and DHEA sulfate, at different concentrations (10^−11^ to 10^−7^ M) also promoted the growth of preantral follicles in a dose-dependent manner [22]. In this study, the AR antagonist hydroxyflutamide but not the aromatase inhibitor fadrozole hydrochloride inhibited the growth response, indicating that estrogens converted from androgens during culture were not responsible for follicular growth.

Androgen hormones are able to promote follicular growth not only during the culture of isolated follicles but also during* in situ* culture. The addition of testosterone (10^−10^ to 10^−7^ M) to the culture medium of fragments of ovarian cortex from bovine fetuses increased the transition from primary to secondary follicles in a dose-dependent manner [85]. In the same study, the addition of estradiol did not promote the same effect; also, in the presence of the AR antagonist flutamide, the positive effect of testosterone on follicular development was completely abolished, indicating that the observed effect was due to the direct action of androgens via AR. The addition of androgens (10^−7^ to 10^−10^ M) to the culture medium of fragments of human ovarian cortex tissue significantly inhibited cell apoptosis [87]. Similarly, the addition of estradiol to the culture medium was unable to reproduce the effect of androgen; this effect was also abolished in the presence of the AR antagonist casodex. These findings suggest a positive effect of androgen hormones on the maintenance of the viability of ovarian tissue during culture, which is exclusively due to a direct androgen action mediated by AR.

In general, the results reported here suggest that, at least under* in vitro* conditions, androgen hormones can promote the growth of ovarian follicles during early stages of development. As observed* in vivo*, high androgen doses can also have a negative influence on follicular development under* in vitro* conditions. The* in vitro* exposure of mouse preantral follicles to androgen concentrations higher than 10^−5^ M induced the precocious luteinization of granulosa cells [90] and also significantly reduced follicular growth and viability [91]. Additionally, under these conditions, the* in vitro* estradiol and progesterone secretion by developing mouse follicles is exacerbated and is associated with reduced oocyte quality and abnormal chromosome distribution on the metaphase plate [92].

## 6. Conclusion

In general, the results compiled in the present review indicate that during the early and intermediate stages of follicular maturation, when AR expression is more pronounced, the androgens locally produced by the developing follicles facilitate the transcription of genes involved in the control of follicle transition from the reserve pool to the growth pool and of genes involved in the promotion of subsequent follicle development. Additionally, because androgens increase the activities of FSH, especially those related to cell proliferation and differentiation, the fall in AR expression in mature follicles reduces the action of androgens; this is possibly a critical event during the processes of follicular selection and atresia. Under* in vitro* conditions, submicromolar androgen doses can have a positive influence on the development of preantral follicles, promoting both survival and growth, especially when combined with the addition of FSH.

## Figures and Tables

**Table 1:** Profile of mRNA expression and of AR protein in the different follicular classes.

	Lenie and Smitz, 2009 [20]		Juengel et al., 2006 [42]		Cárdenas and Pope, 2002 [43]		Salvetti et al., 2012 [41]		Weil et al., 1998 [17]		Chadha et al., 1994 [45]	
Animal	*Rodent *		*Sheep *		*Swine *		*Cattle *		*Non-human primate *		*Human *	
Final Product	mRNA	Protein	mRNA	Protein	mRNA	Protein	mRNA	Protein	mRNA	Protein	mRNA	Protein
Primordial	X	+	+	+	+	X	X	−	−	X	−	X
Primary	X*	+*	+*	+*	+*	X*	X*	−*	−*	X*	−*	X*
Secondary	X*	+*	+*	−*	+*	X*	X*	+*	+*	X*	−*	X*
Initial antral	X*	+*	+*	−*	+*	X*	X*	++*	+*	X*	+/−*	X*
Final antral	X	+	−	−	+	X	X	++	−	X	+/−	X
Oocyte	X	+++	+	+/−	−	X	X	X	−	X	X	X
Theca	X	+	+	−	−	X	X	+	−	X	+/−	X
Granulosa	X	+	++	+	−	X	X	+	+	X	+/−	X

Legend: (X): not done; (−): No expression; (−/+): Little expression; (+): Low expression; (++): Moderate expression; (+++): Abundant expression.

**Table 2:** Effects of the *in vivo* administration of androgen hormones on early follicular development.

Animal	Methodology used	Main results	Reference
Non-human primate	Subcutaneous implants containing testosterone (4 mg/kg animal weight/day) for 3 days, (400 *μ*g/kg animal weight/day) for 10 days, (20 g/kg animal weight/day) for 5 days, or dihydrotestosterone (145 *μ*g/kg animal weight/day) for 5 days.	Increased follicular retrieval, growth, and survival. Increased theca and granulosa cell proliferation.	Vendola et al., 1998 [70]
	Subcutaneous implants containing testosterone (20 *μ*g/kg animal weight/day) or dihydrotestosterone (145 *μ*g/kg animal weight/day) for 5 days.	Increased expression of mRNA for IGF-I and for IGF-I receptor in granulosa and theca cells of growing follicles.	Vendola et al., 1999 [18]
	Subcutaneous implants containing testosterone (20 *μ*g/kg animal weight/day) or dihydrotestosterone (145 *μ*g/kg animal weight/day) for 5 days; testosterone (400 *μ*g/kg animal weight/day) for 3 or 10 days.	Increased follicular retrieval and expression of IGF-I mRNA and of IGF-I receptor in oocytes of primordial follicles.	Vendola et al., 1999 [19]
	Subcutaneous implants containing testosterone (4 mg/kg animal weight/day) for 3 days or testosterone (0.4 mg/kg animal weight/day) for 10 days.	Increased expression of FSH receptor mRNA in granulosa cells of growing follicles.	Weil et al., 1999 [66]

Swine	Intramuscular administration of dihydrotestosterone (60 *μ*g/kg animal weight) for the first 3 days of the early follicular phase or for the last 3 days of the late follicular phase; dihydrotestosterone (6 *μ*g/kg animal weight) administered from the 13th day of the estrous cycle to the next estrus.	Increased ovulatory rate and increased expression of FSH receptor mRNA in periovulatory follicles.	Cárdenas et al., 2002 [73]

Rodent	Administration of a subcutaneous implant containing dihydrotestosterone (83 *μ*g/kg animal weight/day) for 90 days.	Increased follicular retrieval in preantral follicles. Increased potential for *in vitro *development of preantral follicles.	Xue et al., 2012 [74]

**Table 3:** Reproductive results after the *in  vitro* maturation in nonstimulated cycles of patients with polycystic ovary syndrome.

	Maturation (%) (*N*)	Fertilization (%) (*N*)	Cleavage (%) (*N*)	Pregnancy (%) (*N*)
Zhao et al., 2006 [78]	73.7% (632/857)	75.3% (476/632)	91.2% (434/476)	40% (19/47)
Cha et al., 2000 [75]	62.2% (708/1.139)	68% (481/708)	88.1% (266/302)	27.1% (85/23)
Holzer et al., 2007 [77]	68.3% (104/154)	73.3% (71/104)	81.4% (62/71)	50% (6/12)
Zhao et al., 2009 [79]	68.8% (1.753/2.548)	70.28% (1.232/1.753)	90.2% (1.111/1.232)	40% (56/140)
Bos-Mikich et al. 2011 [80]	63% (350/556)	62% (218/350)	95% (208/218)	32% (11/34)

**Table 4:** Effects of the *in vitro* administration of androgen hormones on early follicular development.

Animal	Methodology used	Main results	Reference
Cattle	Culture of ovarian cortex fragments for 10 days in the presence of (i) 10^−10^to 10^−7^ M testosterone, (ii) 10^−7^ M estradiol, and (iii) testosterone in combination with flutamide (AR antagonist).	Testosterone (10^−7^ and 10^−6^ M) promoted an increased transition from primary to secondary follicles, which was inhibited in the presence of flutamide and was not replicated in the presence of estradiol.	Yang and Fortune, 2006 [85]

Human	Culture of ovarian cortex fragments for 24 hours in the presence of FSH in combination with (i) 10^−10^ to 10^−7^ M testosterone, (ii) 10^−10^ to 10^−7^ M dihydrotestosterone, (iii) 10^−10^ to 10^−8^ M estradiol, and (iiii) dihydrotestosterone in combination with casodex (AR antagonist).	Androgens promoted a reduction of ovarian tissue apoptosis which was inhibited in the presence of casodex and was not replicated in the presence of estradiol.	Otala et al., 2004 [87]

Rodent	Culture of isolated preantral follicles for 6 days in the presence of (i) anti-androgen antibody in combination or not with 1 *μ*g/mL androstenedione, (ii) casodex (AR antagonist) in combination with FSH, and (iii) 1 *μ*g/mL dihydroxytestosterone in combination with FSH.	Treatment with an anti-androgen antibody inhibited follicular growth and differentiation; an effect that was reversed by the addition of androstenedione. Treatment with casodex inhibited the positive effect of FSH on follicular growth which was reversed by the addition of dihydroxytestosterone.	Murray et al., 1998 [21]
	Culture of isolated preantral follicles for 6 days in the presence of FSH in combination or not with 1 *μ*g/mL androstenedione.	Treatment with FSH promoted follicular growth and differentiation which were improved when FSH was combined with androstenedione.	Spears et al., 1998 [89]
	Culture of isolated preantral follicles for 4 days in the presence of (i) dihydrotestosterone or testosterone or dihydroxytestosterone or dihydroxytestosterone sulfate (10^−11^ to 10^−7^ M) (ii) in combination or not with hydroxyflutamide (AR antagonist) or FSH.	Treatment with the different androgens promoted a dose-dependent follicular growth which was inhibited by the presence of the AR antagonist. The combination with FSH improved the effect of androgens on follicular growth.	Wang et al., 2001 [22]
	Culture of isolated preantral follicles for 13 days in the presence of (i) 50 *μ*M hydroxyflutamide (AR antagonist) in combination with FSH and (ii) 50 *μ*M bicalutamide (AR antagonist) in combination with FSH.	Follicular growth, production of inhibiting B, and steroids and oocyte maturation were impaired by the addition of both AR antagonists.	Lenie and Smitz 2009 [20]
	Culture of isolated preantral follicles for 13 days in the presence of (i) 20 or 200 nM androstenedione and (ii) testosterone (20 or 200 nM or 2 *μ*M) both in the presence of FSH.	Treatment with both androgens at concentrations of more than 200 nM impaired oocyte maturation.	Romero and Smitz, 2010 [92]
	Culture of isolated preantral follicles for 10 days in the presence of 10^−5^ M androstenedione.	Treatment with androstenedione induced changes in the morphology of granulosa cells compatible with luteinization.	Okutsu et al., 2010 [90]
	Culture of isolated preantral follicles for 12 days in the presence of androstenedione (10^−11^, 10^−9^, 10^−5^ M) in combination with FSH.	Treatment with androstenedione at the dose of 10^−5^ M promoted follicular growth, whereas higher doses impaired follicular development. Treatment with androstenedione impaired oocyte maturation regardless of the dose used.	Tarumi et al., 2012 [91]
